# Factors associated with the first antiretroviral therapy modification in older HIV-1 positive patients

**DOI:** 10.1186/s12981-015-0084-5

**Published:** 2016-01-07

**Authors:** Justyna D. Kowalska, Joanna Kubicka, Ewa Siwak, Piotr Pulik, Ewa Firląg-Burkacka, Andrzej Horban, The Polish Observational Cohort of HIV/AIDS Patients (POLCA) Study Group

**Affiliations:** Hospital for Infectious Diseases, HIV Out-Patient Clinic, Warsaw, Poland; Department for Adult’s Infectious Diseases, Medical University of Warsaw, Warsaw, Poland

**Keywords:** Antiretroviral therapy, Toxicity, Aging, Late presenters, Pharmacovigilance

## Abstract

**Background:**

Rates of first antiretroviral therapy (cART) modifications are high in most observational studies. The age-related differences in treatment duration and characteristics of first cART modifications remain underinvestigated. With increasing proportion of older patients in HIV population it is important to better understand age-related treatment effects.

**Methods:**

Patients were included into this analysis, if being cART naïve at the first visit at the clinic. Follow-up time was measured from the first visit date until first cART modification or 28 February 2013. First cART modification was defined as any change in the third drug component i.e. protease inhibitor (PI), non-nucleoside reverse transcriptase inhibitor (NNRTI), integrase inhibitor or fusion inhibitor. Cox proportional hazard models were used to identify factors related to first cART modification in three age groups: <30, 30–50 and >50.

**Results:**

In total 2027 patients with 14,965 person-years of follow-up (PYFU) were included. The oldest group included 136 patients with 1901, middle group 1202 with 8416 PYFU and youngest group consisted of 689 patients with 4648 PYFU. Median follow-up time was 5.8 (IQR 3.4–9.4) years, median time on first cART was 4.4 (IQR 2.1–8.5) years. 72.4 % of patients started PI-based and 26.1 % NNRTI-based regimen. In total 1268 (62.5 %) patients had cART modification (non-adherence 30.8 %, toxicity 29.6 %). Durability of first cART was the best in patients over 50 y.o. (log-rank test, p = 0.001). Factors associated with discontinuation in this group were late presentation (HR 0.45, [95 % CI 0.23–0.90], p = 0.02) and PI use (HR 2.17, [95 % CI 1.18–4.0], p = 0.01).

**Conclusions:**

Rates of first cART modifications or discontinuation were comparable in all groups; however older patients were significantly longer on first cART regimen.

## Background

Rates of first combination antiretroviral therapy (cART) modifications are high in most observational studies [1–5]. Recent work from the Antiretroviral Therapy Cohort Collaboration (ART-CC) showed that 40.3 % of patients modified first cART with no evidence of decrease in patients starting treatment more recently [1]. While findings from the Swiss cohort confirm this observation, the ATHENA cohort presented almost halved incidence of toxicity-related switches between 1996 and 2010 [4, 5]. Also, as shown by ICoNA cohort, patients who started cART in the ‘recent’ years were less likely to change their initial regimen because of intolerance and/or toxicity [6]. Such decrease is expected with better availability of less toxic and well tolerated treatment options, yet drug-related toxicities remain the leading cause for treatment changes or discontinuations.

With increasing cART utilization, either by earlier initiation or wider access to treatment, it is of highest priority to re-evaluate factors associated with antiretroviral drug toxicities occurrence in clinical settings, especially in relation to toxicities requiring treatment modifications [7–9].

Older age has been linked to increased risk of treatment related toxicities [10–12]. Data from one Italian center showed higher rates of toxicity/adverse events or personal choice discontinuation among patients over 50 years of age [3]. In general the age-related differences in treatment duration and characteristics of first cART modification causes are less evident and remain underinvestigated area in pharmacovigilance [13–17].

Most studies focus on comparing patients over and less than 50 years of age [3, 17]. As a result a patient who is 49 years old would be compared to the one being 51, thus just 2 years older. We believe comparing patients across three age groups may better reflect possible disparities in treatment durability and modifications. The Collaboration of Observational HIV Epidemiological Research Europe study compared age-related treatment responses across different age strata, but information on treatment modifications was not presented [18].

Therefore we have investigated first cART durability and modification causes comparing three age groups.

## Methods

### Study design

This is a retrospective, longitudinal analysis of cART naïve, HIV-infected adults enrolled in Polish Observational Cohort of HIV/AIDS Patients (POLCA) study. POLCA is an observational database cohort where data are collected in real-time from the clinic database. The cohort has been established in 1994 with the purpose of prospective, active observation of HIV-positive patients routinely followed in HIV Out-Patient Clinic, in the Hospital of Infectious Diseases in Warsaw. All data are recorded in the electronic database. On the day of dataset export the cohort included 3087 HIV-positive patients 18 years of age or older with 15,814 person-years of follow-up (PYFU) and 18,269 CD4 cell count and 12,090 HIV RNA measurements. In addition a history of clinical visits, antiretroviral treatment history, demographic characteristics and results of all tests performed in the clinic are available for each patient. Since 2005 a CoDe procedure has been adapted to collect data on death cases in the cohort [19]. A central plasma repository has been established in 2010.

POLCA is an observational cohort therefore patients observed are treated according to standard of care, which is following European AIDS Clinical Society guidelines.

All ethical approvals for the study have been obtained according to Polish regulations.

### Patients and statistical analyses

Patients were included into this analysis, if being cART naïve on the first visit at the clinic. Follow-up time was measured from the first visit date until first cART modification or 28 February 2013. First cART modification was defined as any change in the third drug component i.e. protease inhibitor (PI), non-nucleoside reverse transcriptase inhibitor (NNRTI), integrase inhibitor (ITI) or fusion inhibitor (FI). Due to multifactorial origin and inability to differentiate between medical or administrative/economic decisions, changes in nucleoside reverse transcriptase inhibitors (NRTIs) backbone were not considered as modifications. Simplification of treatment regimens by changing to combination drugs was not considered treatment modification. Discontinuations were considered modifications. A reason of treatment modification was available in the database as specified by patient’s physician via a drop-down selection box available in the clinic’s database.

Persons presenting in care with a lymphocyte CD4 count below 350 cells/µL were considered late presenters according to European Late Presenter Consensus definition [20].

### Statistical analyses

In statistical analyses Chi squared and Kruskal–Wallis tests were used as appropriate, all tests of significance were two-sided. Three age groups were defined taking into accont the age at the first visit in the clinic: <30, 30–50 and >50 years old. Cox proportional hazard models were used to identify factors associated with treatment modification. A multivariable model was developed including all variables tested in univariate models. The potential predictors of treatment modifications tested in univariate models were: age, gender, HIV acquisition risk group, the lowest (nadir) lymphocyte CD4+ count, anti-HCV and anti-HBc total antibodies status, baseline haemoglobin level, ARV group (PI, NNRTI, ITI or FI) and late presentation. Confidence interval (CI) of 95 % was accepted. All analyses were performed using SAS version 9.1 (SAS Institute, Cary, NC, USA).

## Results

A total of 2027 patients with 14,965 PYFU were included into the study. The group of oldest patients included 136 patients with 1901 PYFU, middle group 1202 with 8416 PYFU and youngest patients’ group consisted of 689 patients with 4648 PYFU.

Median follow-up time was 5.8 (IQR 3.4–9.4) years, median time on first cART was 4.4 (IQR 2.1–8.5) years. In total 1467 (72.4 %) patients started PI-based cART, 529 (26.1 %) NNRTI-based and 31 (1.5 %) other cART regimen.

Patients over 50 years of age were more advanced in HIV disease with lower first and nadir lymphocyte CD4 count. They were also more likely to have most recent HIV RNA level below 50 copies/ml, to have anti-HBc total antibodies positive test result and less likely to have anti-HCV antibodies positive tests result. Median age in this group was 55.7 (53.0–60.0) years (Table 1).

**Table 1:** Baseline characteristics of all three age groups

	Patients less than 30 y.o. N = 689, N (%)	Patients 30–50 y.o., N (%)	Patients over 50 y.o. N = 136, N (%)	P value*
Female	217 (26.3)	169 (14.1)	15 (11.0)	<0.0001
MSM	268 (38.9)	505 (24.9)	48 (35.3)	<0.0001
Positive anti-HCV antibodies	231 (33.5)	361 (30.0)	20 (14.7)	<0.001
Positive anti-HBc total antibodies	144 (20.9)	376 (31.3)	60 (44.1)	<0.0001
Positive VDRL	98 (14.2)	216 (18.0)	21 (15.4)	0.17
Late presenters (CD4 <350)	293 (42.5)	661 (55.0)	87 (64.0)	<0.0001
First cART				0.79
PI	491 (71.3)	880 (73.2)	96 (70.6)	
NNRTI	188 (27.3)	303 (25.2)	38 (27.9)	
Any cART modification	414 (60.1)	771 (64.1)	83 (61.0)	0.20
Last HIV RNA <50** *opies/ml*	543 (64.7)	952 (79.2)	126 (86.9)	<0.0001
	Median (IQR)			
Age in years	26.6 (23.8–28.4)	35.9 (32.4–40.3)	55.7 (53–60)	–
Haemoglobin level	14.1 (13.0–15.1)	14.0 (12.6–15.1)	13.3 (11.5–14.2)	<0.0001
First CD4 (cells/µl)	416 (268–570)	317 (163–184)	240 (80–480)	<0.0001
Nadir CD4 (cells/µl)	244 (143–337)	173 (69–274)	166 (61–301)	<0.0001
Last CD4 (cells/µl)	504 (367–673)	476 (315–638)	488 (356–631)	0.012
First HIV RNA (log10 copies/ml)**	4.33 (3.56–4.87)	4.38 (3.40–4.99)	4.43 (3.02–5.01)	0.54
Follow-up time in years	5.1 (3.2–8.3)	6.1 (3.4–10.1)	5.2 (3.6–9.2)	<0.0001
Time on cART in years	3.4 (1.7–7.3)	4.8 (2.4–9.0)	4.8 (2.0–8.5)	<0.0001
Time on first cART in years	1.4 (0.5–2.8)	2.0 (0.8–3.8)	2.0 (0.7–4.1)	<0.0001

* Chi square test

** Abbott RealTime HIV-1 Test

PI based therapy was most frequent first regimen prescribed in all three groups and the youngest patients were shorter on first cART regimen (Table 1).

In total 1268 (62.5 %) patients had treatment modification. The most common reasons were non-adherence (30.8 %) and treatment related toxicities (29.6 %). Approximately 6 % of modifications had no specified reason (unknown). Among different causes of treatment modification cART failure was more frequent in oldest patients (p = 0.09), whereas non-adherence in both younger groups (p < 0.001). Toxicities were comparably frequent reason for cART change in all age groups. Death occurred in 9.6 % of oldest, 3.6 % of middle age and 2.4 % of youngest patients, Table 2.

**Table 2:** Comparison of reasons for treatment modification between older and younger patients

Reason for treatment modification	Patients less than 30	Patients from 30 to 50 y.o.	Patients over 50	P value*
Total	441 (100)	771 (100)	83 (100)	–
cART toxicity	136 (32.8)	232 (30.2)	30 (36.1)	0.41
cART failure	20 (4.8)	50 (6.5)	9 (10.8)	0.09
Non-HIV related disease	8 (1.9)	17 (2.2)	2 (2.4)	0.89
Non-adherence	116 (28.0)	262 (34.0)	13 (15.7)	<0.001
Pregnancy	12 (2.9)	1 (0.1)	0 (0.0)	<0.0001
Migration	21 (5.1)	55 (7.1)	3 (3.6)	0.15
Death	10 (2.4)	28 (3.6)	8 (9.6)	0.006
Other	65 (15.7)	80 (10.4)	13 (15.7)	0.07
Unknown	26 (6.3)	46 (6.0)	5 (6.0)	1.00

* p value < 0.001

**Fig. 1:**
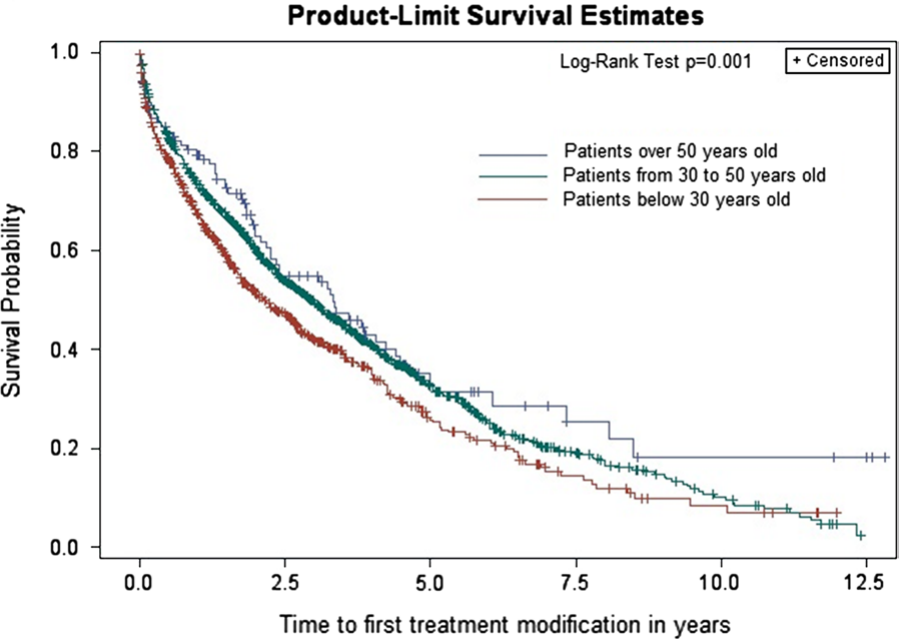
Kaplan-Meier survival curve for the time to treatment modification after starting first cART regimen

The durability of first cART was better in older patients (log rank test, p = 0.001). Fig. 1 presents Kaplan–Meier survival curve of time to treatment modification after starting first cART regimen stratified by age group.

Factors significantly associated with treatment modification in oldest patients’ group were baseline hemoglobin level, positive anti-HBs total antibodies status, late presentation and PI use. However in multivariable Cox proportional hazard models analyses only late presentation [HR 0.45, (95 % CI 0.23–0.90), p = 0.02] and PI use [HR 2.17, (95 % CI 1.18–4.0), p = 0.01] remained statistically significant (Table 3).

**Table 3:** Univariate and multivariate hazard ratios of the risk of treatment modification in the group of patients over 50 years old

	Unvariate				Multivariate^a^			
	P value	Hazard ratio	95 % hazard ratio confidence limits		P value	Hazard ratio	95 % hazard ratio confidence limits	
Gender								
Male	0.14	0.61	0.31	1.81	0.37	0.71	0.33	1.51
Female	–	1.00	–	–	–	1.00	–	–
HIV risk group								
MSM	0.08	0.62	0.36	1.06	0.09	0.60	0.33	1.09
Heterosexual	–	1.00	–	–	–	1.00	–	–
IDU	0.33	1.41	0.70	2.84	0.58	1.29	0.52	3.21
Other	0.44	0.70	0.28	1.74	0.50	0.72	0.28	1.85
Unknown	0.37	0.72	0.35	1.48	0.52	0.78	0.37	1.66
Nadir CD4 value								
Per 100 increase	0.62	1.05	0.87	1.25	0.18	0.84	0.66	1.08
Age in years								
Per 1 year increase	0.87	1.00	0.97	1.04	0.73	1.01	0.97	1.05
Anti-HCV Ab								
Positive	0.22	1.43	0.80	2.54	0.33	0.65	0.28	1.53
Negative	–	1.00	–	–	–	1.00	–	–
Unknown	0.55	0.77	0.33	1.81	0.29	0.60	0.23	1.56
Anti-HBc Ab								
Positive	0.50	1.2	0.7	2.06	0.30	1.36	0.76	2.45
Negative	–	1.00	–	–	–	1.00	–	–
Unknown	0.29	1.37	0.76	2.46	0.36	1.37	0.70	2.71
Baseline hemoglobin								
Per 1 g/dL increase	0.05	1.02	1.0	1.04	0.14	1.01	0.995	1.04
ARV group								
PI	0.03	1.79	1.06	3.02	0.01	2.17	1.18	3.98
NNRTI	–	1.00	–	–	–	1.00	–	–
ITI	0.99	0	0	0	0.99	0	0	–
FI	0.99	0	0	0	0.99	0	0	–
Late presentation								
Yes	0.03	0.61	0.39	0.96	0.02	0.45	0.23	0.90

^a^Adjusted for all above

Factors significantly associated with treatment modification in middle age group were gender, HIV risk group, nadir CD4 + count, baseline hemoglobin level, positive anti-HCV and anti-HBs total antibodies status, late presentation and cART group. In multivariable Cox proportional hazard models analyses male gender (HR 1.50, [95 % CI 1.03–2.18], p = 0.034), injecting drug use as HIV infection mode (HR 0.59, [95 % CI 0.40–0.87], p = 0.007), nadir CD4+ count (HR 1.44, [95 % CI 1.32–1.57], p < 0.001), unknown anti-HBc total status (HR 0.67, [95 % CI 0.52–0.86], p = 0.002), cART group as ITI (HR 24.7, [95 % CI 9.7–63.4], p < 0.001) and FI (HR 2.73, [95 % CI 1.47–5.04], p < 0.001) remained statistically significant (Table 4).

**Table 4:** Univariate and multivariate hazard ratios of the risk of treatment modification in the group of patients 30 years old to 50 years old

Parameter	Unvariate				Multivariate			
	P value	Hazard ratio	95 % hazard ratio confidence limits		P value	Hazard ratio	95 % hazard ratio confidence limits	
Gender								
Male	<0.001	1.95	1.40	2.73	0.034	1.50	1.03	2.18
Female	–	1.00	–	–	–	1.00	–	–
HIV risk group								
MSM	<0.001	1.71	1.31	2.23	0.09	1.29	0.96	1.72
Heterosexual	–	1.00	–	–	–	1.00	–	–
IDU	0.002	0.57	0.41	0.81	0.007	0.59	0.40	0.87
Other	0.11	0.55	0.27	1.15	0.12	0.56	0.27	1.17
UNKN	0.74	1.07	0.71	1.61	0.94	1.02	0.67	1.55
Nadir CD4 value								
Per 100 increase	<0.001	1.46	1.37	1.56	<0.001	1.44	1.32	1.57
Age in years								
Per 1 year increase	0.50	0.99	0.98	1.01	0.29	0.99	0.97	1.01
Anti-HCV Ab								
Positive	<0.001	0.40	0.31	0.53	0.24	0.81	0.56	1.16
Negative	–	1.00	–	–	–	1.00	–	–
Unknown	0.45	0.88	0.63	1.23	0.06	1.44	0.98	2.11
Anti-HBc Ab								
Positive	<0.001	0.60	0.48	0.76	0.07	0.79	0.62	1.02
Negative	–	1.00	–	–	–	1.00	–	–
Unknown	<0.001	0.59	0.47	0.74	0.002	0.67	0.52	0.86
Baseline hemoglobin								
Per 1 g/dL increase	0.09	1.01	1.00	1.03	0.57	0.99	0.95	1.03
ARV group								
PI	0.35	1.11	0.89	1.38	0.18	1.16	0.93	1.46
NNRTI	–	1.00	–	–	–	1.00	–	–
ITI	<0.001	22.9	9.14	57.5	<0.001	24.7	9.66	63.4
FI	<0.001	3.85	2.11	7.02	0.001	2.73	1.47	5.04
Late presenter								
Yes	<0.001	0.65	0.54	0.78	0.79	1.05	0.83	1.33

In the group of youngest patients gender, HIV risk group, nadir CD4+ count, baseline hemoglobin level, positive anti-HBs total and anti HCV antibodies status were significantly associated with treatment modification in univariate analyses. After adjustment for confounding variables only nadir CD4+ count remained significant factors decreasing the risk of modification (HR 0.89, [95 % CI 0.81–0.98], p = 0.017), (Table 5).

**Table 5:** Univariate and multivariate hazard ratios of the risk of treatment modification in the group of patients less than 30 years old

Parameter	Unvariate				Multivariate^a^			
	P value	Hazard ratio	95 % hazard ratio confidence limits		P value	Hazard ratio	95 % hazard ratio confidence limits	
Gender								
Male	<0.001	0.71	0.58	0.86	0.33	0.88	0.69	1.13
Female	–	1.00	–	–	–	1.00	–	–
HIV risk group								
MSM	0.01	0.68	0.51	0.92	0.17	0.77	0.55	1.11
Heterosexual	–	1.00	–	–	–	1.00	–	–
IDU	0.01	1.41	1.08	1.84	0.09	1.31	0.96	1.81
Other	0.50	1.24	0.66	2.34	0.59	1.19	0.62	2.29
Unknown	0.70	1.08	0.72	1.62	0.82	1.05	0.69	1.61
Nadir CD4 value								
Per 100 increase	0.002	0.88	0.81	0.96	0.017	0.89	0.81	0.98
Age in years								
Per 1 year increase	0.07	0.97	0.94	1.0	0.07	0.97	0.93	1.00
Anti-HCV Ab								
Positive	<0.001	1.62	1.32	1.98	0.58	1.08	0.81	1.44
Negative	–	1.00	–	–	–	1.00	–	–
Unknown	0.13	1.3	0.93	1.82	0.37	1.20	0.81	1.77
Anti-HBc Ab								
Positive	0.01	1.37	1.07	1.75	0.36	1.14	0.87	1.49
Negative	–	1.00	–	–	–	1.00	–	–
Unknown	0.34	1.11	0.89	1.39	0.86	1.02	0.80	1.31
Baseline hemoglobin								
Per 1 g/dL increase	0.01	0.93	0.88	0.98	0.71	0.99	0.92	1.06
ARV group								
PI	0.87	1.02	0.82	1.27	0.49	0.92	0.74	1.16
NNRTI	–	1.00	–	–	–	1.00	–	–
ITI	0.97	0	0		0.96	0	0	–
FI	0.56	0.71	0.23	2.25	0.68	0.78	0.25	2.48
Late presenter								
Yes	0.87	0.98	0.81	1.19	0.20	0.86	0.69	1.08

^a^Adjusted for all above

## Discussion

In this study we present that patients over 50 years of age had much better treatment durability as compared to other age groups. Factors significantly increasing the risk of cART modification in this group of patients were PI use and late presentation. Although rates of first cART modifications were comparable in both groups, treatment discontinuation due to its toxicity or failure was more frequent among oldest patients, whereas due to non-adherence in younger patients. This is to our knowledge the first study to present such age-related differences in the frequency of the discontinuation causes.

Co-infections and co-morbidities are more frequent in patients over 50, thus toxicities more likely to occur and more likely to be the cause of treatment modifications [21, 22]. However, in our work rates of modifications related to non-HIV diseases were only slightly increasing across age groups, being in general a rare cause of switching. The additional challenge for starting cART in older persons is polypharmacy [23], however in our analyses we were not able to control for this factor.

Interestingly PI based cART regimens were preferable in all groups and in older patients were associated with increased risk of treatment modification. Higher PI utilization may reflect the need to initiate treatment immediately due to low CD4+ lymphocyte count and when the genotyping test result is not yet available. Although the primary mutations are infrequent in Poland it is generally preferred to receive genotyping test result before starting treatment [24]. This could lead to the increased utilisation of PIs in patients initiating cART in our study.

We found that older patients had better viral suppression, defined by the proportion of patients with latest HIV RNA level below 50 copies/ml. Studies investigating effects of age on treatment response are inconsistent and does not provide insight into failure related modifications across age groups [17, 18, 25]. According to COHERE the probability of virological response was higher for persons aged 50–59 as compared to 30–39 years old [18].

In our study late presentation was 20 % higher in older patients and only in this group of patients significantly associated with lower risk of treatment modification. Studies of large HIV-positive populations present lower lymphocyte CD4+ count and shorter time to AIDS for older patients, which is well in line with higher rate of late presenters among older patients in most observational cohorts [14]. In a large nationwide study in the United Kingdom late presenters were more likely to be over 50 years of age and contributed to 67 % of all patients over 50 [26]. This findings are showing an urgent need for improved testing strategies, as increasing utilization of HIV testing among older persons may result in earlier HIV diagnosis [27–29].

The presented lower rate of non-adherence related treatment modification in older patients is well in line with data published by other cohorts [30].

There are some limitations, which need to be highlighted. We focused exclusively on the third drug in regimen. We estimate that differences presented in our work would be even more profound, should the NRTI group switching be included. Due to small numbers we were not able to investigate further the effects of specific antiretroviral drugs and the drug-related risk of ART discontinuation [31]. We have not investigated the impact of cART modifications on mortality due to small number of deaths in the investigated group of patients. Whether and to what extent treatment modifications influence the general treatment outcome measured as mortality remains especially important in older patients, thus should remain in future studies focus [16].

It needs to be mentioned that our study’s background may vary from other countries and/or regions in terms of demographics, preferred first line treatment regimens and timing of treatment initiation.

Although persons over 50 years old are contributing to approximately half of HIV-positive population treatment modifications in this group of patients are rarely investigated [32]. Studies should focus on this important area in order to better adjust HIV care to patient needs and age related health problems. Such approach could significantly contribute to further decrease in morbidity and mortality of HIV-positive population.

## Conclusions

Although rates of first cART modifications in our study were comparable between age groups, we have shown a substantial differences in reasons for treatment modification. Older patients were also significantly longer on first cART regimen. In multivariate Cox proportional hazard models factors significantly increasing the risk of cART modification in older patients included late presentation and PI use. Our findings present age-related differences in terms of treatment tolerability warranting the need for further studies in this area, as well as opening a discussion about age-adjusted approach in HIV treatment strategies.
